# Microstate in rats’ EEG: a proof of concept study

**DOI:** 10.1038/s41398-025-03702-y

**Published:** 2025-11-21

**Authors:** Vaclava Piorecka, Cestmir Vejmola, Petra Peskova, Marek Piorecky, Stanislav Jiricek, Vlastimil Koudelka, Inga Griskova-Bulanova, Tomas Palenicek

**Affiliations:** 1 Dept. of Biomedical Technology, Faculty of Biomedical Engineering, Czech Technical University in Prague, Kladno, Czech Republic; 2 Clinical Research Program, National Institute of Mental Health, Klecany, Czech Republic; 3 Psychedelic Research Centre, National Institute of Mental Health, Klecany, Czech Republic; 4 Third Faculty of Medicine, Charles University in Prague, Prague, Czech Republic; 5 Sleep and Chronobiology Research Centre, National Institute of Mental Health, Klecany, Czech Republic; 6 Institute of Computer Science, Czech Academy of Sciences, Prague, Czech Republic; 7 Faculty of Electrical Engineering, Czech Technical University in Prague, Prague, Czech Republic; 8 Institute of Biosciences, Vilnius University, Vilnius, Lithuania; 9 Translational Health Research Institute, Vilnius University, Vilnius, Lithuania

**Keywords:** Physiology, Predictive markers

## Abstract

The electroencephalogram (EEG) reflecting brain activity may be characterised through brief periods of stable neural activity patterns that recur over time and are referred to as microstates. Microstates are related to a range of cognitive processes, and their analysis has become an increasingly popular tool for studying human brain function. While microstates have been extensively studied in humans, their presence and characteristics in animal models have yet to be as thoroughly investigated. This study aims to address this gap by detecting and characterising microstates in EEGs of rats collected using a superficial electrode system corresponding to homological areas of the human 10–20 system. Specifically, we demonstrate the presence of microstates in rats’ EEGs, i.e., those that may be captured by the same metrics as in humans. We identified five microstate EEG maps in rats, explaining 71% of the variance in our dataset (*N* = 30). The explained variance, mean temporal coverage values (0.2), and average duration (0.26 s) are comparable to the human-derived EEG microstates. Via a source localisation technique, the cingulate cortex, precuneus, and insula were found to be associated with the microstates’ temporal dynamics. Among the microstates that showed a broadband character, we also found those that showed an association with the theta and alpha bands. These findings have important implications for the use of microstates as a preclinical tool for investigating brain functions, detecting new biomarkers of brain diseases, and translating this knowledge to humans.

## Introduction

Electroencephalography (EEG) is a non-invasive technique that allows the recording of brain activity with high temporal resolution. In the past few decades, EEG microstate analysis has become a popular tool for investigating the functional organisation of the human brain [1, 2].

Microstates are brief periods of stable topographical configurations of scalp voltage maps lasting a few hundred milliseconds (40–120 ms). Typically, around or more than 70% of the variance in the human EEG signal during resting state (no active task) is attributed to four to seven prototypical microstate classes, denoted as A, B, C, D, and E, F, G [3]. Each microstate class is characterised by distinct topographic appearances [3–5] and associated functional correlates [3]. It is assumed that microstates represent the brain’s functional states, during which various brain regions communicate in a coordinated manner [6]. Indeed, several studies have shown that intracortical sources of various microstates spatially correspond to the hubs of fundamental resting state networks [7, 8]. Therefore, the temporal features of the microstates, such as their average duration in milliseconds, frequency of occurrences per second, and their percentage contribution to the EEG signal, may serve as sensitive indicators of various momentary mental states and stable trait characteristics [9–12]. Additionally, these temporal characteristics may serve as potential biomarkers for mental and neurological disorders, approaching a valid tool for pre-clinical and clinical research.

Given this emerging significance of microstates, it is striking that they have so far been overlooked in studies on animal models, which would not only allow an in-depth understanding of microstates through feasible manipulations (pharmacological, genetic, behavioural, or neural manipulations such as optogenetics or deep-brain electrode stimulation) but also stand as a valuable tool in translational research. To the best of our knowledge, only four up-to-date studies have reported the analysis of microstates in rodent models. Mégevand et al. observed that somatosensory-evoked responses in mice may be characterised by a series of distinct cortical map configurations, each one remaining stable for a given period of time and then quickly changing into a new configuration in which it remained stable again [13]. Two other studies focused on the identification of microstates for the estimation of the spatiotemporal properties of neuronal ensembles in local field potential (LFP) recordings. The authors reported the existence of “mesoscale” microstates that exhibit high spatial coherence, temporal stability, and frequency-specific modulation. These microstates were modulated by behavioural states, such as movement or exploration [14], and by the depth of anaesthesia [15]. Most recently, Boyce et al. [16] studied EEG microstate dynamics during resting wakefulness in mice from multi-EEG surface recordings and demonstrated that local cortical neural assembly activity coordinates with global brain dynamics during wakefulness but not during sleep.

Although the above studies suggested that microstates are present in rodents like in humans, none used a homologous electrode system and rigorously described microstate parameters using appropriate metrics to render the method translationally usable. Here, we analysed signals from 30 rats recorded using 21 electrodes placed in regions homologous to the human 10–20 EEG system and adopted analysis approaches used in human research to achieve this. We employed several criteria to determine the optimal number of microstates, and, following Mishra et al. [14], utilised surrogate data analysis to contrast the global explained variance (GEV) parameter between randomly shuffled data and actual EEG recordings. We described the characteristics of the optimal microstates, including their topographies, duration, occurrence, and GEV values. We also explored their correlation structure in spatial and temporal domains. Additionally, following human EEG microstate studies [17, 18], we aimed to determine the brain areas involved in the generation of microstates. This knowledge is crucial for the translational utility of the microstate approach from humans to animal models and for understanding the neural basis of brain function across species.

The primary aim of our study was to explore the presence and characteristics of microstates in rat EEG. Specifically, we tested the hypothesis that microstates can be reliably detected in rat EEG signals recorded with a homologous electrode system comparable to the human 10–20 system (H1). Given the exploratory nature of the study, we focused more on broader expectations, which are formulated qualitatively. We anticipated that microstate parameters in rats (e.g., topographies, duration, occurrence, and explained variance) might show patterns broadly comparable to those reported in humans (E1). We also explored whether microstates in rat EEG could originate from brain regions analogous to those observed in humans (E2). These exploratory expectations highlight the potential translational relevance of the method.

## Materials and methods

### Data collection

All the experiments were conducted on adult male Wistar rats (SPF animals; Velaz, Czech Republic) weighing 280–300 g. A total of 30 animals were used. Each animal was tested only once. Access to water and a standardised diet was ad libitum. Ethical approval was given by the National Committee for the Care and Use of Laboratory Animals, CZ, and European Union guidelines and principles (86/609/EU) were adhered to.

**Fig. 1:**
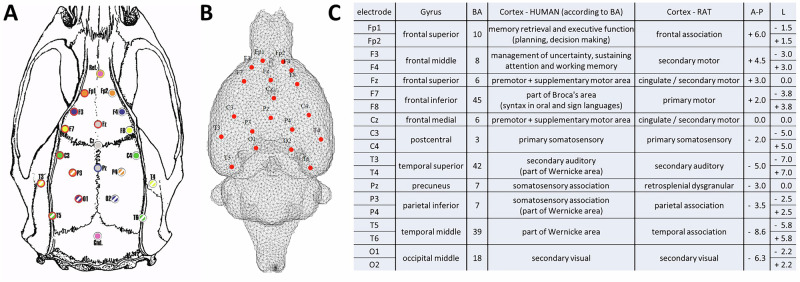
Electrode layout and coordinate correspondence between the rat and human systems. **A** Electrode layout shown on the rat’s skull. **B** Brain mesh model used for forward modelling. **C** The table showing the electrodes with their coordinates and locations in the original 10–20 system distribution in humans, with homologous regions of selected coordinates for the rat system. BA, Brodmann areas; A-P, antero-posterior; L, lateral. The coordinates are given in millimetres, according to [19].

The rats were stereotactically implanted with 21 gold-plated electrodes (Mill-Max) under general isoflurane anaesthesia (2.5% concentration). The electrodes were implanted onto the surface of the cortex in homologous frontal, parietal, and temporal regions of the right and left hemispheres. The coordinates were designed to homologically and functionally match the human 10–20 electrode system. Coordinates were taken from the Paxinos rat brain atlas [19]. Their positions are listed in Fig. 1. The reference electrode was implanted above the olfactory bulb, and the ground electrode was placed subcutaneously in the occipital region. All the electrodes were fixed to the skull with dental cement. The whole procedure took on average 45 min. Postoperatively, the rats were treated with ketoprofen (5 mg/kg, s.c.) and housed individually (to prevent biting of the implant) and handled daily to habituate them to the manipulations. One day before recording, a connector was mounted to the electrodes under short-term anaesthesia and fixed with dental cement.

The experiments were conducted during the daytime, between 7:30 and 13:00, 7 days after surgery. Approximately 15 min before the experiment began, the animals were connected to the EEG system in their home cages. Subsequently, a 100 min recording was taken while the rats were allowed to move freely around the cage. EEG data were collected as part of a study examining the effects of pharmacological substances (for details, see [20]). Recordings commenced 10 min before substance administration and continued thereafter. For the present analysis, only the 10 min baseline period was considered. Raw EEG signals were acquired using a BioSDA09 standard 32-channel digital EEG amplifier (M&I Ltd., Prague, Czech Republic) at a sampling rate of 250 Hz and stored on a PC hard disk for offline processing and analysis. In parallel with the EEG data recording, two types of behavioural activity (active behaviour/inactivity) were scored by an experienced observer. The animals were handled for a few seconds if a suspicion of sleep was observed (i.e., the animals did not move and tended to close their eyes).

### Preprocessing

The first part of the data preprocessing was performed by an expert. The data were pre-processed in BrainVision Analyzer 2 software (Brain Products GmbH). Firstly, the data were manually inspected, and all artifacts were discarded. Then, the data were segmented by markers indicating behavioural (in)activity, and only epochs corresponding to behavioural inactivity were extracted as a model of resting state EEG data. Segments shorter than 2 s were excluded from further analysis. The remaining data were concatenated and treated as one continuous time series. The data were then exported to be analysed in Matlab. The second part of the data preprocessing was performed using the EEGlab toolbox [21]. Here, the EEG recordings were re-referenced to averages and filtered using a 1–40 Hz two-way band-pass FIR filter with 2000 filter coefficients.

### Microstate analysis

The microstate analysis was performed on 2 min segments of broadband (1–40 Hz) EEG. The microstates were calculated at all peaks of the global field power (GFP) [1, 22] by the agglomerate hierarchical clustering algorithm (AAHC algorithm) [23, 24]. The microstate analysis was performed using the EEGLAB plugin for microstates, version 1.2, developed by Thomas Koenig [25]. The analysis was performed for different microstates ranging from 2–10. The global average topographies were used for backfitting to the individual EEG recordings.

All typical microstate parameters (coverage, occurrence, duration, and GFP) were calculated for microstate description. The strength of the average global activation during a given microstate $k$ was defined by its average GFP of all EEG samples assigned to microstate $k$:

$${{GFP}}_{k}=\frac{1}{{N}_{k}}\mathop{\sum }\limits_{n\in k}^{{N}_{k}}{{GFP}}_{n}$$

where ${N}_{k}$ is the number of samples assigned to the cluster $k$ [26].

Each microstate duration was defined as the time in milliseconds during which consecutive original maps belonged to the same microstate class. This time began and ended halfway between the last map of the previous microstate and the first map of the next. Occurrence was defined as the number of times a microstate class appeared per second across all analysis epochs. Coverage was defined as the proportion of the total analysis time occupied by a given microstate class, expressed either as a fraction (0–1) or as a percentage (%) [27].

The transition probabilities between the microstate classes were extracted as the asymptotic behaviour of transitions between microstates (i.e., the likelihood of switching between different microstates) [28]. The expected transitions were calculated and are defined by the following equation:

$${ExpTM}\left(k1,k2\right)=\frac{\frac{{Occ}\left(k1\right)}{{MeanOcc}}\cdot \frac{{Occ}\left(k2\right)}{{MeanOcc}}}{1-\frac{{Occ}\left(k1\right)}{{MeanOcc}}},$$

where $k1,k2$ represent the microstate number, ${Occ}$ represents the occurrence, and ${MeanOcc}$ represents the mean occurrence [25].

The above-mentioned parameters have proven to be effective markers for distinguishing different brain states in the case of human EEG [29].

### Optimal Number of Microstate Classes

For an estimation of the optimal number of microstate classes, several indexes were utilised based on human studies, i.e., the Krzanowski-Lai (KL) criterion [23, 30], the cross-validation criterion [4], or the metacriterion [7], which consists of several individual criteria. A list and description of the parameters that optimise the number of microstates may be seen in the supplemental part, see Appendix A.

The global explain variance (GEV), the percentage of data variance explained by a given set of microstate maps [23, 31]) was calculated. The GEV value for a specific microstate map with the label $k$ is defined by the following equation [32]:

$${{GEV}}_{k}=\frac{\mathop{\sum }\limits_{i}{{GFP}}_{i}^{2}{\cdot }{C}_{{ik}}^{2}\cdot {\delta }_{{k,L}_{i}}}{{\sum }_{i}\,{{GFP}}_{i}^{2}},$$

where ${GF}{P}_{i}$ is the global field power (defined as the standard deviation of the instantaneous EEG topography), ${\delta }_{k,{L}_{i}}$ is the Kronecker delta, i.e., ${\delta }_{k,{L}_{i}}=1$ for ${L}_{i}=k$ and ${\delta }_{k,{L}_{i}}=0$ otherwise, and ${C}_{{ik}}^{2}$ is the squared covariance between the instantaneous EEG topography and candidate microstate map. The total global explained variance (totGEV) is the sum of the GEV values over all microstate maps [32]:

$${totGEV}=\mathop{\sum }\limits_{k}\,{{GEV}}_{k.}$$

### Generation of shuffled data

The surrogate data were generated to facilitate statistical permutation testing. The process involved generating a random vector of time positions for shuffling, repeated 10 times for each EEG record. Subsequently, the beginnings and ends of each electrode time series were swapped, resulting in temporal shuffling of the data. This method is based on the study by Mishra et al. [14], where a similar technique is used for the validation. The surrogates were then used for the GEV calculation, and a statistical analysis of the GEV values was performed to identify if there was a difference between the microstate models of the surrogates and the EEG data. If the microstate model specifically captures EEG brain states, then there should be significantly higher GEVs observed on the brain data compared to the surrogates.

### Spatial similarity of microstates

To quantify the spatial similarity between the identified microstate topographies, the Pearson correlation coefficient [33] was calculated. This was done to ensure that the polarity of topographies was ignored and the absolute value of Pearson’s correlation was taken.

### Source localisation of microstates

Following the human EEG microstates studies [17, 18], we aimed to determine the brain sources associated with the temporal evolution of individual microstates, considered as a representation of brain EEG activity. For this purpose, we generally followed the source localisation pipeline introduced in our previous study [34]. The only difference from the previous study was that a 19-electrode cortical EEG system (instead of 12) was utilised to define the forward model. To estimate the sources, we applied the eLORETA inverse algorithm [35], implemented in the FieldTrip toolbox [36], to whole 2 min-long segments extracted from each EEG recording as described in subsection 2.2. Firstly, three time series corresponding to three orthogonal directions were estimated for each source position. Secondly, three time series were projected onto the first principal component, which was further considered an EEG source time series for a given source position. To study the frequency specificity of the underlying brain sources as in [17], each EEG source time series was further band pass filtered (two-pass Butterworth filter, order 3) into typical human EEG frequency bands: Delta ($\delta$, 2–4 Hz), Theta ($\theta$, 4–8 Hz), Alpha ($\alpha$, 8–12 Hz) and Beta low and mid-range ($\beta$, 12–20 Hz). Subsequently, the signal envelope (${BLP}$) was calculated by applying the Hilbert transform to each time series and taking the absolute value. The rationale behind associating the microstate time series with frequency-specific signal envelopes is two-fold. Firstly, the literature supports the idea that the underlying sources of human EEG microstates are frequency-specific [17]. Secondly, the signal envelope of a broadband signal is naturally driven by oscillations with the highest power, i.e., the $\delta$ frequency band, which may result in overlooking other frequency-specific information when associating with the microstate time series. To determine the brain sources associated with each of the five microstate topographies, we utilised and adapted a regression approach from [18]. For each subject $s$ and the microstate $k$, a regressor ${B}_{s,k}$ was derived as:

$${B}_{s,k}=|{{EEG}}_{s}{{Map}}_{k}|,$$

where ${EE}{G}_{s}$ is the EEG data matrix with dimensions (time samples x number of electrodes), and ${Ma}{p}_{k}$ is a single-session map of a microstate $k$. Note that for each frequency-specific signal envelope, the microstate regressor was the same. The regressor used for the source localisation analysis represents microstate dynamics in a continuous manner [37] and does not strictly conform to the assumption of discrete switching between states. However, as demonstrated in [38], clear microstate switching is prominent during periods of high GFP, while it becomes less prominent during segments with lower GFP. Importantly, a continuous regressor is also more suitable for comparison with BLP fluctuations, which typically exhibit slow temporal dynamics.

Furthermore, to eliminate the influence of global power signal fluctuation, we included two other regressors into a linear model: GFP and global map dissimilarity (GMD) as in [18]. Therefore, for each source $s$, microstate $k$, and frequency band $f$, we estimated the parameters of the following linear model:

$${{BLP}}_{s,f}={\beta }_{0}+{\beta }_{1}{B}_{k}+{\beta }_{2}{GFP}+{\beta }_{3}{GMD}+{\epsilon }_{k}.$$

Finally, for each microstate $k$ and frequency band $f$, we performed a group-level statistical analysis on the ${\beta }_{1}$ parameter to determine where in the brain and in which frequency band ${B}_{k}$ is related to the source-space BLP. The Monte Carlo permutation statistical analysis with the threshold-free cluster enhancement (TFCE) method to correct for multiple comparisons [39] was performed utilising the FieldTrip toolbox [36] to determine where ${\beta }_{1}$ differs from zero ($\alpha$ = 0.001, two-sided test). To associate clusters where the difference of ${\beta }_{1}$ from zero is mostly pronounced to rat brain areas, we also coregistered an anatomical rat brain atlas from [40] to label each source-space position to a corresponding rat brain anatomical area. The obtained statistical maps were subjected to two summary statistics. Firstly, we calculated the total cluster size by simply dividing all sources surviving TFCE correction by the total number of sources, which represents the spatial as well as frequency specificity of the given microstate association with source-space BLP. Secondly, a correlation matrix of each frequency-specific microstate statistical map (TFCE correction masked) was created to evaluate intra- as well as inter-microstate frequency-dependent statistical source map similarities.

## Results

### Optimal number of microstate classes

**Fig. 2:**
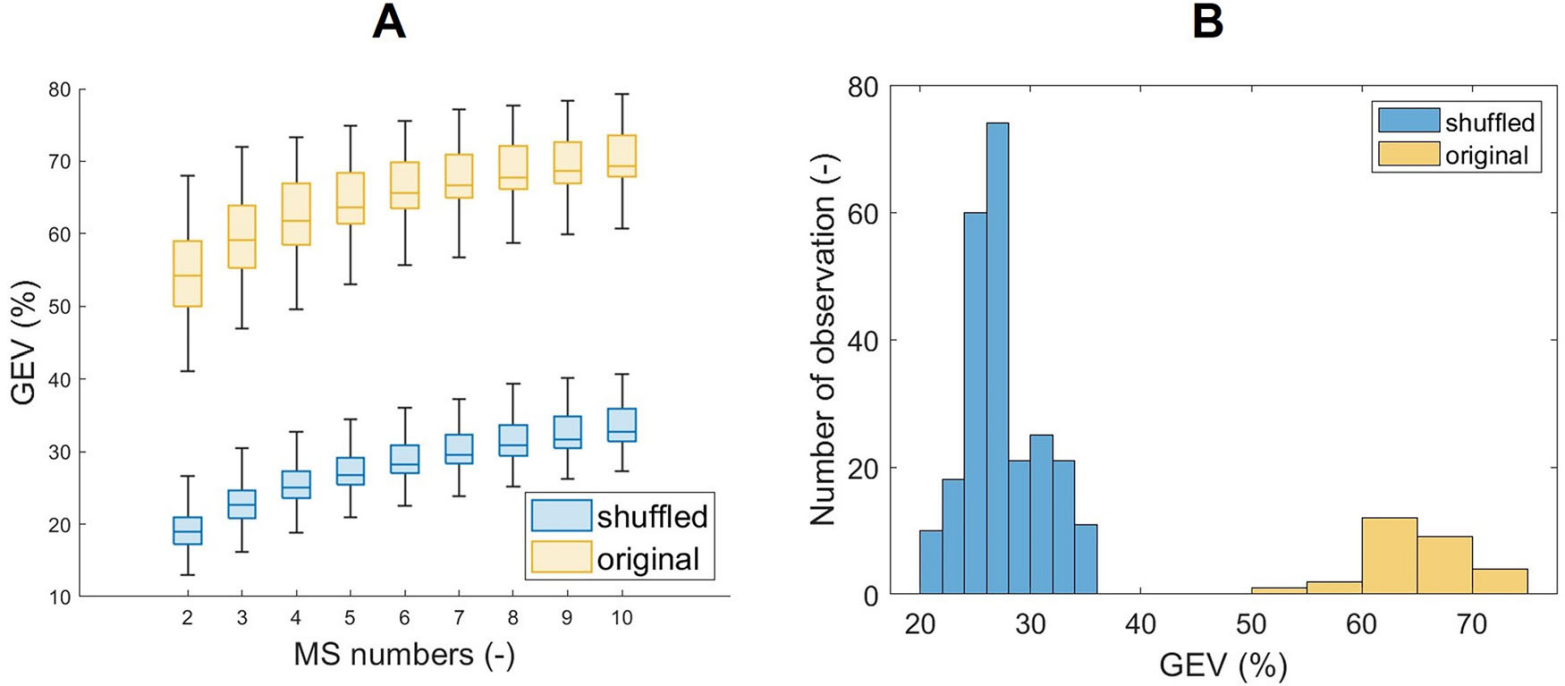
Comparison of Global Explained Variance (GEV) between the original and shuffled datasets. **A** depicts boxplots of Global Explained Variance (GEV) values for the original dataset (yellow) and the temporally shuffled dataset (blue), across microstate models comprising 2–10 states. **B** presents the histogram of GEV values for the 5-state solution, representing the distribution of the data underlying the corresponding boxplot in (**A**). The approximately Gaussian form of the histogram indicates that the distribution of GEV values can be reasonably assumed to follow normality. The histogram also reflects the sizes of the data sets.

The results of the estimation of the optimal number of microstates utilising CV, KL, Davien Bouldin, Dunn, Frey, and Van Groenewould, dispersion, and metacriterion methods showed substantial differences when estimating the number of microstate classes. As the KL method exhibits insensitivity to the number of electrodes [41] and is frequently used in human studies [42–49], the outcome of KL was set as the preferred choice for the current study, resulting in an optimal number of microstate classes set at five. More details on the assessment of microstate classes are provided in the Supplementary material, see Appendix A and B and Fig. 2.

### Validation on shuffled data

The GEV parameter was calculated for both the original broadband and shuffled data. The outliers were excluded from both groups. An outlier is a value of more than three scaled median absolute deviations (MAD) from the median. Boxplots of the mean GEV and histograms for each number of microstates (from 2–10) are plotted in (Fig. 2A).

Normality testing using the Kolmogorov-Smirnov test revealed that neither the whole shuffled dataset nor the shuffled dataset for the five optimal microstates followed a normal distribution (*p*-value $<$ 0.001). The same non-normal distribution pattern was observed for the original whole dataset and the original dataset for the five optimal microstates (*p*-value $<$ 0.001). The two-sample Kolmogorov-Smirnov test confirmed (at an alpha level of 0.05) that the data did not stem from the same distribution (*p*-value $<$ 0.001). Statistically significant differences between the distributions were identified for both the entire datasets and the five optimal microstates.

The GEV values for the original broadband dataset were, on average, 64.81 ± 7.06 (mean ± STD) for the microstates from 2–10 and 64.72 $\pm$ 4.94% for five microstates specifically. In contrast, for the temporally shuffled dataset, the average GEV values were 30.10 ± 7.31 (mean ± STD) for the microstates from 2–10 and 29.55 ± 6.00 for the five microstates correspondingly. The significant decrease in GEV values after temporal shuffling suggests that the outcomes of the microstate analysis are non-random.

### Microstate description

**Fig. 3:**
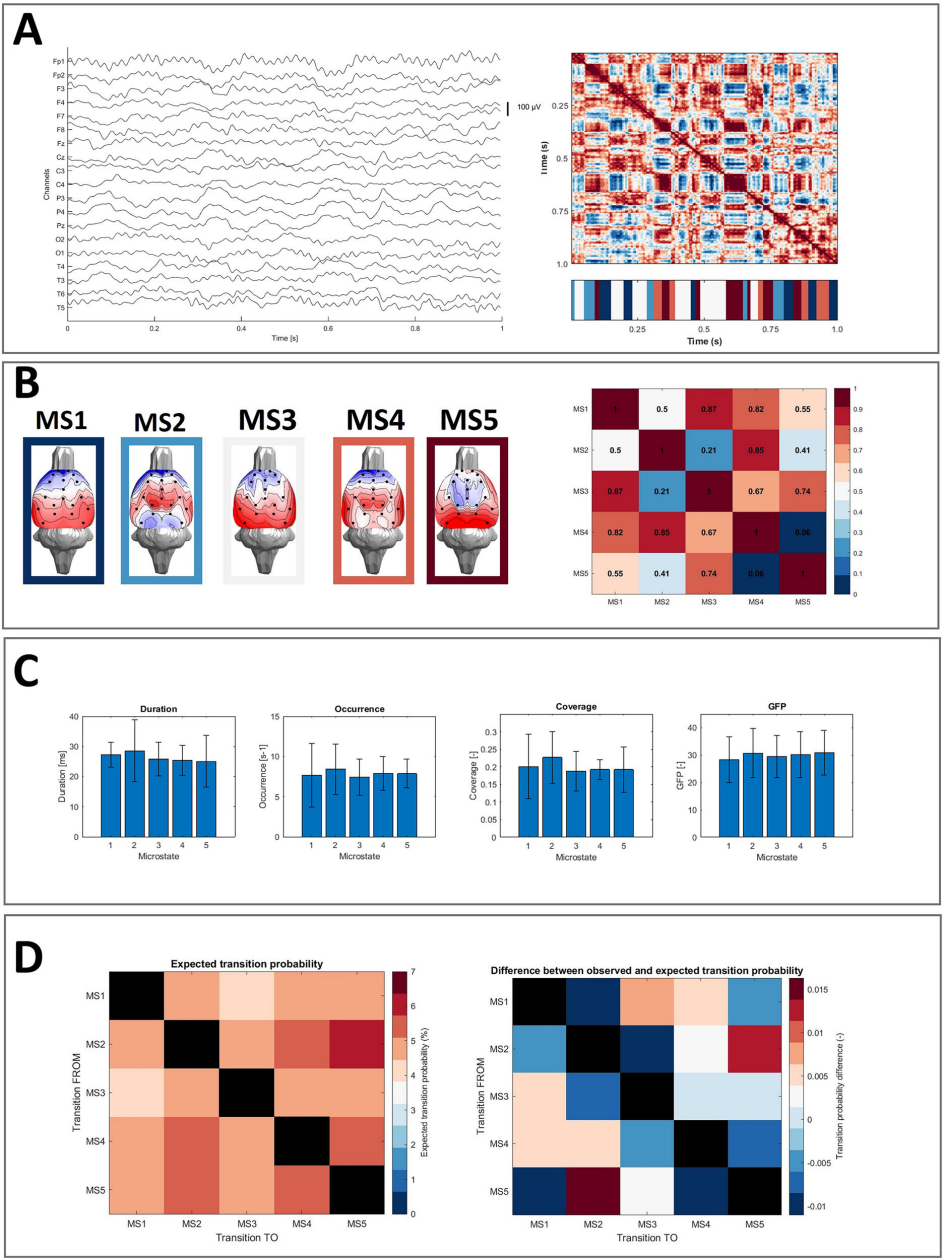
The results of the microstate analysis on rats’ EEG. **A** represents the pre-processed rats’ EEG (left), the spatial correlation of the topographic maps between time samples (right top), and the resulting microstates sequence. **B** depicted the resulting five microstate topologies and the spatial correlation between the microstate topographies. Please note that the colour frames are in line with the microstate sequence. **C** describes the classical microstate parameters, concretely, their mean and standard deviation values for each microstate. **D** transition probabilities between microstates: the left matrix shows expected probabilities normalized by occurences (in percentage) and the right matrix illustrates the difference between the observed and expected transitions based.

A topographical representation of the five extracted microstate classes and their spatial correlation coefficients are presented in Fig. 3B. MS1 and MS3 display similar anterior-posterior topography; MS2 is centred on the midbrain and further spreads to the parietal posterior regions. MS4 is represented by a both-sided temporal activation connected on a mid-line, and MS5 is represented by a central locus. The topographies and spatial correlation plots for the microstates from 2–10 are presented in the Supplementary material for information purposes, see Appendix C.

Details of the classical parameters of microstate analysis for the five microstates are presented in Fig. 3C. As can be seen, the average coverage of the microstates was at around 0.20, and the duration was measured at around 0.26 s. Transition probabilities between the extracted microstates are plotted in Fig. 3D. Microstates exhibit distinct transition patterns, with transition probabilities varying between states. The transition probabilities are bidirectionally higher between MS2 and MS5, and between MS2 and MS4. The corresponding numerical values in tabular form, along with the results of the statistical evaluation of the differences between the expected and observed values, are presented in the Supplementary Material (Appendix D).

### Source localisation of microstates

**Fig. 4:**
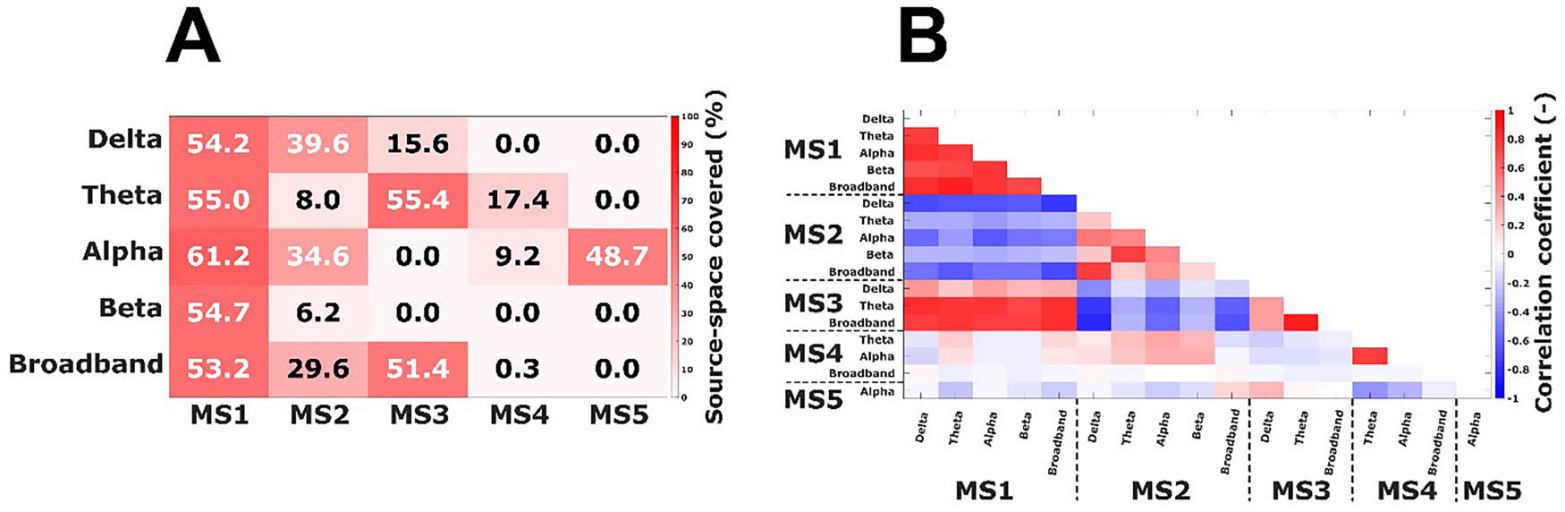
Source localisation summary and correlation analysis of microstates. **A** The activation level of microstates in the brain (as a percentage of sources included within clusters to the total number of sources). **B** Pearson correlation analysis of frequency-dependent source maps (both within and between microstates). Note that rows and columns corresponding to non-significant maps are omitted.

We compiled group-level statistical maps representing the association between each microstate time course and frequency-specific source-space BLP signal fluctuations. Figure 4A shows the percentage of the total cluster size from the total amount of sources for each microstate and frequency band. Figure 4B shows pair-wise correlation coefficients of each frequency-specific microstate map. Rows and columns corresponding to statistical maps not having significant activations are omitted.

**Fig. 5:**
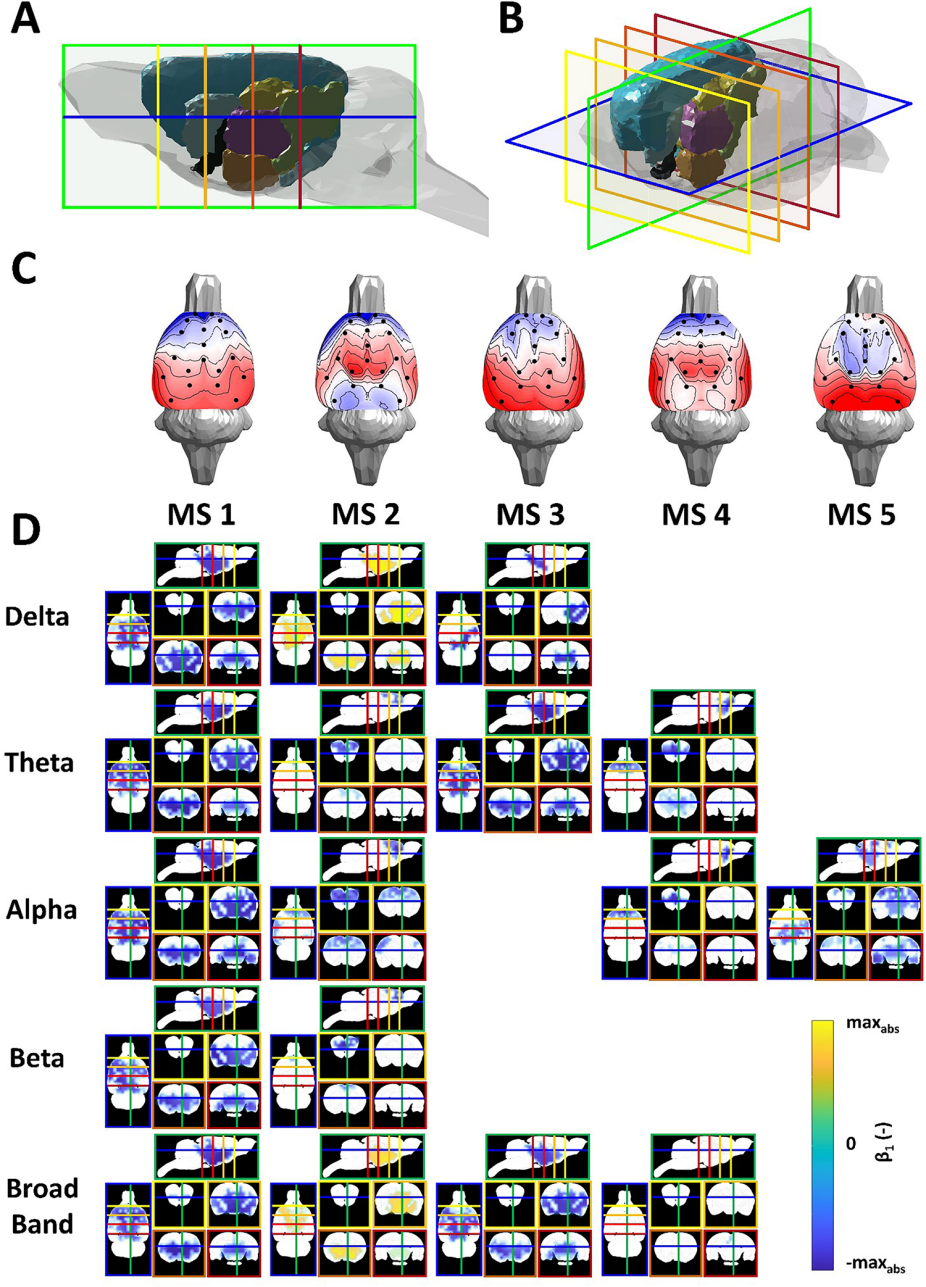
Sources of microstates. (**A,** **B**) 3D model of the rat brain showing selected cutting planes for source presentations from a right (**A**) and top right (**B**) view. The planes are: horizontal (−6 mm ventro-dorsally, framed in blue), sagittal (+0.5 mm laterally, framed in green), and four coronal sections (+3.5 mm anteroposteriorly, framed in yellow; 0 mm, in light orange; −3.5 mm, in dark orange; −7 mm, in red), coordinates refer to bregma according to [19]. Structures shown: Isocortex (light blue), Pallidum (black), Hippocampus (golden yellow), Hypothalamus (coral red), Diencephalon (orchid pink), Midbrain (light green), Striatum (light cyan) (**C**) Topographic maps of the five microstates. **D** Group cluster permutation statistic values (*β*_1_, *p* $< \,$0.001) interpolated on the rat brain anatomical MRI scan shown in the respective planes with the microstate classes in columns and the frequency bands in rows. The colour framing of the section images corresponds to the colour labelling of the sections in Fig. 5B.

Figure 5 shows the actual source maps. It is possible to observe that MS1 is not frequency-specific, but its time series is mostly associated with source activity in each frequency band. Spatially, the clusters cover more than half of the source space, and the statistical maps seem to correlate strongly between frequency bands. MS1 extends over the entire thalamus, basal ganglia, and insular cortex over all frequency bands. In the case of MS2, the association with source activity is more pronounced in the delta and alpha frequency bands as well as the broadband. The broadband statistical map is more similar to the delta statistical map than to the other frequency-specific maps. All the MS2 statistical maps correlate negatively to all the MS1 statistical maps. In the delta band, or rather, in the broadband, MS2 occupies a similar space as MS1 but is localised slightly lower, extending to the ventral pallidum and hypothalamus. The theta to beta band shows the greatest activity frontally, i.e., the prelimbic cortex and frontal association cortex, and then in the primary and secondary auditory cortex. Interestingly, MS3 is widely associated with the source-space activity in the theta and broadband frequency bands, which are also strongly intercorrelated. MS3 patterns generally do not correlate to any of the statistical maps of MS2 but correlate positively to MS1 statistical maps, as MS3 replicates MS1 in its sources; see Fig. 5. In the delta band, however, MS3 defines a narrow band of the granular insular cortex, retrosplenial dysgranular cortex, and periaqueductal grey activity. MS4 expresses alpha and theta activity in the frontal association area, insular and infralimbic cortex, and its statistical maps are highly correlated. Conversely, MS4 maps are less similar to the other microstate statistical maps. Finally, MS5 only has an alpha band-specific source-space pattern that is, again, less similar to the patterns of other microstates than MS1 to MS3. It extends over large areas of the primary and secondary auditory, entorhinal, dysgranular, and granular insular cortex.

## Discussion

Our results indicate that EEG signals from freely moving rats may be largely represented by short-lasting voltage topographies that remain stable for brief periods before rapidly switching to a new configuration. Such “momentary brain electric field configurations” persist for tens of milliseconds and tend to cluster into a limited number of recurring classes, the same as the microstates in humans. By applying various criteria to determine the optimal number of microstates and choosing the KL method, we identified five microstate classes. Validation against shuffled data confirmed that these microstates represent non-random temporal patterns, as demonstrated by significantly higher GEV in the original dataset compared to shuffled data. Each of the five microstates displayed distinct scalp topographies and classical parameters (duration, occurrence, coverage, and GFP), with transition probabilities suggesting certain preferred transitions. Source localisation revealed specific frequency-dependent activation patterns for each microstate, highlighting both cortical and subcortical regions and underscoring the translational potential of microstate analysis for understanding large-scale brain dynamics in animal models.

In humans, a small set of prototypical microstate maps (commonly four canonical classes labelled A–D) is traditionally identified across individuals [8, 31]. However, the development and implementation of more sensitive criteria has resulted in a recommendation not to strictly adhere to the number four [18]. Recent studies have demonstrated that the optimal number of microstates describing resting-state EEG varies from four to seven. We found that rats also exhibit repeatable microstate topographies across subjects, suggesting that the basic phenomenon of quasi-stable brain states is conserved. Using the Krzanovski-Lai criterion that is insensitive to the number of electrodes [41] and frequently used in human studies [50], we identified five microstate maps explaining 71% of the variance, somewhat less than the variability explained in human studies, which is commonly around 80% [3]. Although the actual values of the explained variance are not directly comparable between rats and humans, one possible explanation for observing a lower explained variance in rats is that cortical EEG recordings are unaffected by the blurring effects of skull and scalp tissues. This results in a richer signal that may be less effectively captured by the same number of microstates as in scalp EEG studies. It is also worth noting that most human research is conducted with the participants’ eyes closed. Studies investigating EEG microstates during eyes-open conditions have shown that, while the topography and relative proportions of parameters remain largely unchanged, there is typically a decrease in overall measures such as GEV, duration, and time coverage [51, 52]. Consequently, the fact that our rats’ eyes were open may have contributed to the observed findings.

In the classical human 4-map arrangement, microstate map A exhibits a left-right orientation, map B a right-left orientation, map C an anterior-posterior orientation, and map D a fronto-central maximum. Even if more cluster maps are selected, these four canonical maps seem to consistently dominate the data across different age ranges, conditions (e.g., sleep and hypnosis), and pathological states [1]. Here, the estimated rat microstates were also characterised by distinct dominant topographies (Fig. 3). Although the spatial patterns of rat microstates do not directly mirror human microstates (likely due to differences in cortical anatomy and electrode coverage), the observed spatial correlation between topographic maps across the time samples further confirms the presence of microstates in rats. Fig. 3A shows clear temporal clusters of topographically similar EEG activity. MS1 exhibits a frontal to occipital topography and highly correlates to MS3 (pronounced occipital activity) and MS4 (fronto-central and both-sided temporal activation); MS2 was characterised by central activation and demonstrated a strong spatial correlation to MS4. Finally, MS5 resembled a centro-parietal locus of activity. The identified microstates showed a high number of spatial inter-correlations, a finding commonly reported in human data [18]. Although none of the rat microstates show a lateralised form, as is true for human microstates A and B, based on spatial similarity, it is possible to infer that rat microstate 1 is most similar to human microstate C [4, 7], in humans associated with control of cognitive processes [8, 53]. Rat microstate 5 may then represent human microstate D [4, 7], which in humans is associated with the vigilance level [51, 53, 54].

EEG microstate dynamics have emerged as sensitive biomarkers of brain dysfunction, with consistent alterations reported across a wide range of neuropsychiatric conditions, including schizophrenia [55], mood disorders [56], and ADHD [57]. These brief, quasi-stable patterns of brain activity reflect a coordinated and large-scale network function and show disorder-specific changes in their temporal structure and topography. For instance, Koenig et al. [58] demonstrated microstate alterations in schizophrenia that correlated with symptom severity, while other disorders exhibit different but systematic deviations from normative patterns. These findings suggest that microstates reflect key aspects of brain network function and its disruption in disease. Michel and Koenig (2018) [7] noted that disturbances of mental processes manifest as changes in the temporal dynamics of specific microstates, highlighting their diagnostic and prognostic potential. Extending microstate analysis to rats, as is described here, enables controlled investigation of pathological and treatment-related changes. If rat microstate metrics parallel those in human disorders, then they may serve as preclinical biomarkers for probing disease mechanisms and evaluating novel therapies. Their reliability and reproducibility [50] further support their translational value.

Among the four rodent studies capturing microstate-like activity, only Mishra et al. [14] reported results in a traditional way that allows for parameter comparison. However, they used microelectrode LFP recordings, targeting some kind of mesoscale microstates, while we employed a superficial electrode system targeting areas homologous to those studied in humans. Mishra et al. provided GEV values against surrogates of 0.71 for the prefrontal cortex and striatum and 0.64 for the ventral tegmental area, which align closely with our findings. Although, like Mishra et al. [14], we observed a significant increase in GEVs compared to the surrogate data, Jajcay et al. [59] showed that in the case of surrogates based on vector regression models and Fourier transforms, the microstates were not statistically distinguishable from them. Moreover, Pascual-Marqui et al. [60] showed that microstate properties may be effectively captured by a multivariate cross-spectrum EEG model. Therefore, caution should be taken when interpreting microstate findings in relation to surrogate data.

The mean temporal coverage values (0.23 for the prefrontal cortex (PFC), 0.23 for the striatum (STR), and 0.26 for the ventral temporal area (VTA) in Mishra et al. [14] are also consistent with our result of 0.20. However, a key difference emerged in the average duration of microstates: 26.99 ms in our study compared to 68.04 ms for PFC, 70.30 ms for STR, and 65.41 ms for VTA in Mishra et al., i.e., values that are more similar to those found in human research (60–120 ms). Despite this difference, our results had lower standard deviations, and our durations still fell within the predicted range for human microstates (20.7– 106.8 ms) [50]. The shorter durations in our study likely led to a higher microstate occurrence rate, approximately twice that observed by Mishra et al. Importantly, the choice of four microstates in their study may have contributed to the differences in the reported parameters, as variations in the number of microstates are known to influence both duration and occurrence rates [7]. As indicated by the transition probabilities between microstate classes, certain patterns of the activity were more stable than others. For example, the bidirectional transitions from MS2 to MS5 and MS2 to MS4 were the most frequently occurring ones, showing that the dynamics between microstates are likely to be unique, as in humans [28, 51]. However, this aspect remains difficult to compare due to a lack of normative data on microstate transition probabilities in humans.

We estimated the neuronal generators of five resting-state-like topographies using a recently developed source localisation approach [34] and temporal general linear modelling inspired by the study of [18]. This analysis identified several areas of activation shared across three microstate classes (MS1-MS3), with each of the five microstates consistently linked to distinct frequency band-specific brain regions. The overlap in brain areas across microstate source spaces likely explains the partial spatial similarities in scalp potential maps (i.e., resting-state-like topographies). These shared regions include areas along the anterior and posterior medial axes, the insula, and the superior parietal cortex. Custo and colleagues also identified a set of brain regions active across most microstate networks in human microstates [18]. These regions, including the anterior and posterior cingulate cortices, precuneus, superior frontal cortex, supramarginal gyrus, dorsal superior prefrontal cortex, and insula, are key hubs frequently highlighted in studies of structural and functional brain networks [61–63].

Previous human studies have linked EEG microstates to fMRI BOLD signals to associate large-scale brain networks with specific microstates [8, 64]. Microstate A was connected to the auditory network, microstate B to the visual network, microstate C to the salience network, and microstate D to the attention network, with corresponding brain regions. While many studies have identified large-scale rat brain networks, recent debate on the overlap and subdivision of fMRI resting-state networks suggests caution when linking microstates to specific brain functions based solely on fMRI correlations [7]. In [65] the authors provided a simultaneous trimodal PET–MR–EEG imaging study resulting in a higher correlation between the microstate neural generators and receptor availability of GABA_A_ (inhibitory) compared to mGluR5 (excitatory) receptors and glucose metabolism. At the same time, the receptor availability of GABA_A_ was found to be related to default mode and salient networks specifically. In our study, MS1 showed the most prominent activity within the thalamus, basal ganglia, and insular cortex; MS2 shared similar locations; however, it was centred lower, also reaching the ventral pallidum, hypothalamus, prelimbic, frontal association, and auditory cortices. MS3 also shared regions with MS1, including the granular insular and retrosplenial cortices with periaqueductal grey. MS4 was specific for the frontal association, insular and infralimbic cortices, and MS5 for the auditory, entorhinal, dysgranular, and granular insular cortices.

Milz and colleagues [17], unlike the others [8, 64], provided conclusive results on the interrelationship between EEG microstate classes and spectral power characteristics, and showed that human microstates are driven predominantly by alpha activity. In [65] a predominance of inhibitory processes during the resting state was shown and a possible relation between microstate generators and EEG beta activity was suggested. Our results show that the rat cortical surface topography of microstate classes appears to be determined by intra-cortical sources of specific frequency bands. While MS1 and MS2 seem to be generated by activity within the broad frequency range, MS3 seems to be low-frequency specific, MS4 is typical for the theta-alpha frequency range, and finally, MS5 is purely alpha band specific.

By employing the rat cortical EEG source localisation technique [34], we are able to analyse the results at the level of specific brain regions, further enhancing the interpretability of the obtained microstate maps. However, it is crucial to recognise the inherent challenges associated with the EEG inverse problem [66]. Additionally, since the electrodes are primarily situated on the top part of the brain cortex, this setup limits the resolution for detecting activity in deeper brain structures. Consequently, the reduced accuracy in localising signals from deep brain regions has to be considered.

## Conclusion

The growing interest in non-invasive tools for evaluating brain function in various disorders highlights the need for translational methods applicable to both humans and animals. In this study, we aimed to identify and thoroughly describe microstates in the resting-state EEG of freely moving rats, which may be comparable to those observed in humans. Resting-state EEG microstates are a widely used and promising technique for assessing brain function in both healthy and pathological conditions in humans, but this approach has been largely overlooked in animal studies.

This study provides the first comprehensive identification and description of microstates in the resting-state EEG of freely moving rats, with findings that resemble human-like microstates. By using a classical microstate analysis pipeline and source localisation techniques, we identified five microstate EEG maps in rats, explaining 71% of the variance in our dataset. These results align with human studies and suggest that microstates in rats may be linked to brain networks similar to those in humans, speculatively also to cognitive control and vigilance. The shared brain regions identified across the microstates in our source localisation analysis, including key hubs like the cingulate cortex, precuneus, and insula, further highlight the translational potential of this approach. Although care must be taken when linking microstates to specific brain functions, this study opens new avenues for cross-species research on brain function using microstate analysis.

## Supplementary information

Appendix

## Data Availability

The data that support the findings of this study are not publicly available due to privacy and ethical restrictions, but are available from the corresponding author upon reasonable request.
