# Supplementary material for: Microstate in rats’ EEG: a proof of concept study
Source: Transl Psychiatry. 2025 Nov 21;15:494. doi: 10.1038/s41398-025-03702-y (PMC12638752; doi:10.1038/s41398-025-03702-y)
Supplement: Supplementary file 1 — Appendix [file 41398_2025_3702_MOESM1_ESM.docx]

# Appendix

# Appendix A Description of the Criterion

The meta-criterion combines several criteria, each operating on a slightly different principle of determining the optimal number of microstates. The interquartile range (IQR) and the interquartile mean (IQM) are calculated based on all the criterion estimates [1]. The meta-criterion may include, for example, the following criteria: Davies and Bouldin [2], KL [3], Dunn’s [4,5], Frey and Van Groenewoud’s criterion [5,6], and dispersion criterion [7].

The most commonly used criterion is cross-validation (CV), which was introduced in Pascual-Marqui et al. [8]. This measure is related to residual noise, and the objective is to obtain a low value of CV, which is defined by the following equation:

$$CV=\delta^{2}\cdot\left( \frac{C-1}{C-K-1} \right)^{2},$$

where $\delta^{2}$ defines the estimation of variance of the residual noise, $C$ is the number of the EEG channels, and $K$ is the number of clusters [7, 8]. However, because CV is a ratio between GEV and the degrees of freedom for a given set of template maps, this criterion is highly sensitive to the number of electrodes in the montage [9].

The KL criterion was introduced in Krzanowski and Lai 1988 [3], as a means of selecting how many clusters to use based on the dispersion measure. High values of KL usually indicate an optimal number of clusters [7]. It works by first calculating a quality measure of segmentation, termed dispersion ($W$), which is defined by the following equations:

$$W_{k}=\sum_{k}^{K} \frac{S_{k}}{2\cdot N_{k}},$$

where

$$S_{k}=\sum_{n}^{N} \sum_{n'}^{N'} \parallel x_{n}-x_{n'}\parallel^{2},$$

for $l_{n}=k\wedge l_{n'}=k$ . The variable $S_{k}$ represents the sum of the pair-wise distance between all maps of the given cluster $k$ and $N_{k}$ is the number of maps for cluster $k$. The following equations then calculate the KL criterion:

$$KL\left( K \right)=\left| \frac{DIFF\left( K \right)}{DIFF\left( K+1 \right)} \right|,$$

$$DIFF\left( K \right)=\left( K-1 \right)^{\frac{2}{C}}W_{K-1}-K^{\frac{2}{C}}W_{K},$$

where $W_{k}$ represents the dispersion as described in the previous equations. The $KL$ criterion attains large values when an elbow in the $W_{k}$ curve occurs. [7, 9]

The boxplot graphs of observed criteria are depicted in Figure A1.


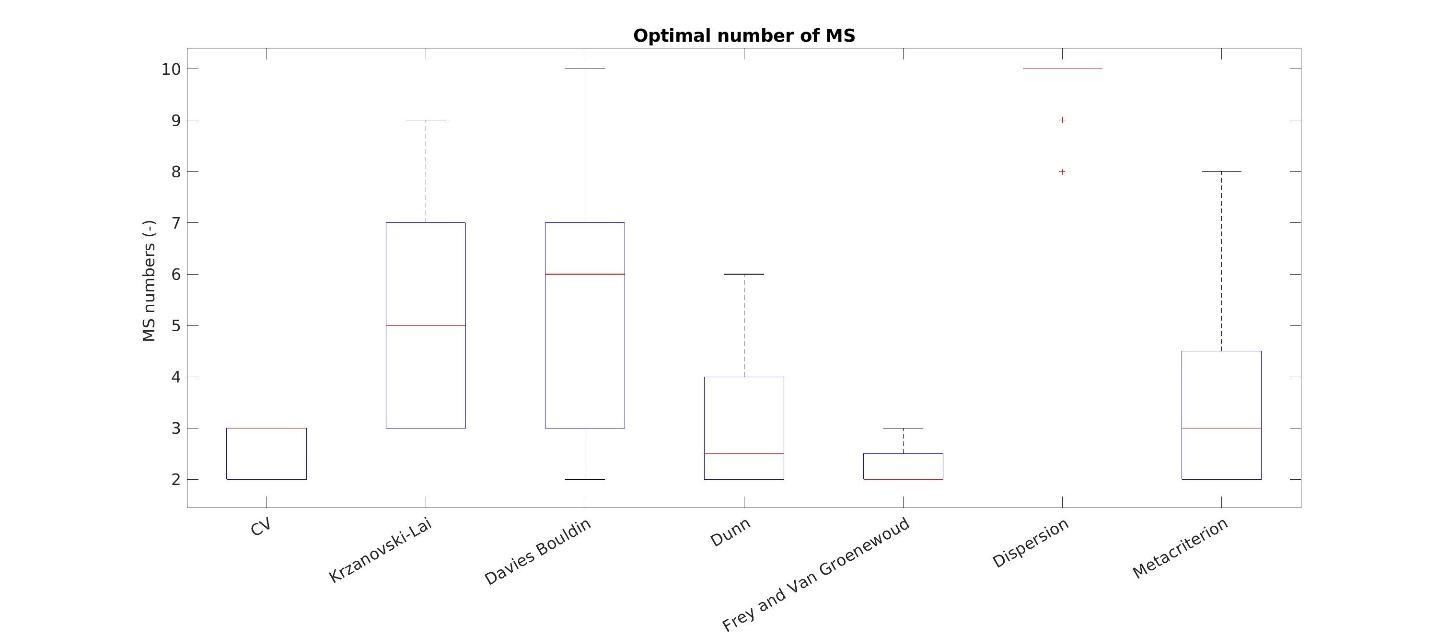


**Figure A1** *Optimal number of microstates based on several criteria.*

# Appendix B Changes in CV and GEV

The CV and GEV parameters were calculated for the total number of microstates from 2 to 10. The mean value and standard deviation were calculated for both parameters and are depicted in Figure B1.


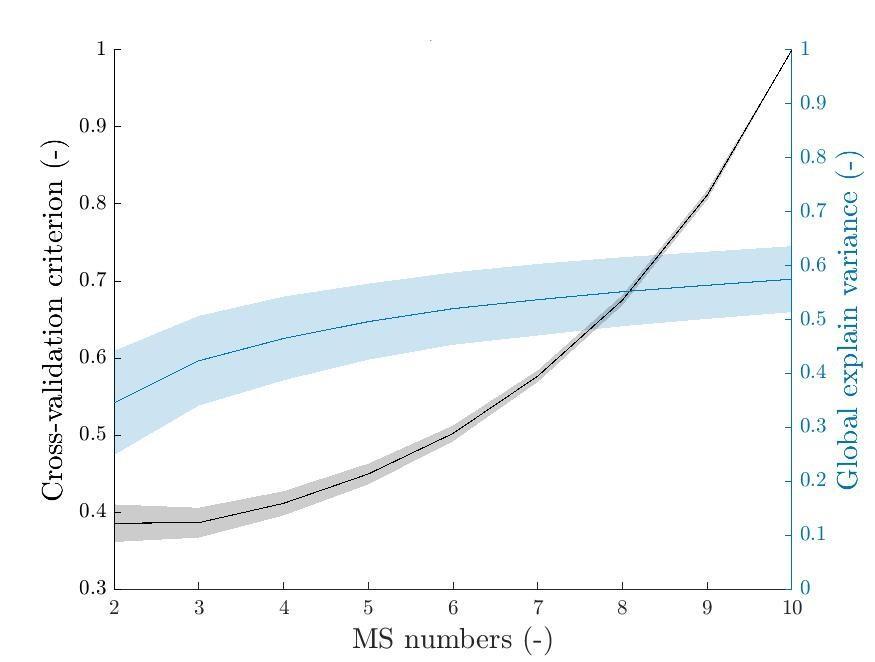


**Figure B1** *The cross-validation (in black) and GEV (in blue) parameters across subjects are depicted here. The mean and standard deviation are presented for each variable.*

# Appendix C Microstate Topography Across the Different Microstate Numbers

This appendix reports the microstate topographies for the different numbers of microstates and the spatial correlations between them, see Figures C1-C9. Microstate analysis was performed as described in Section [2](#bookmark=id.30j0zll).


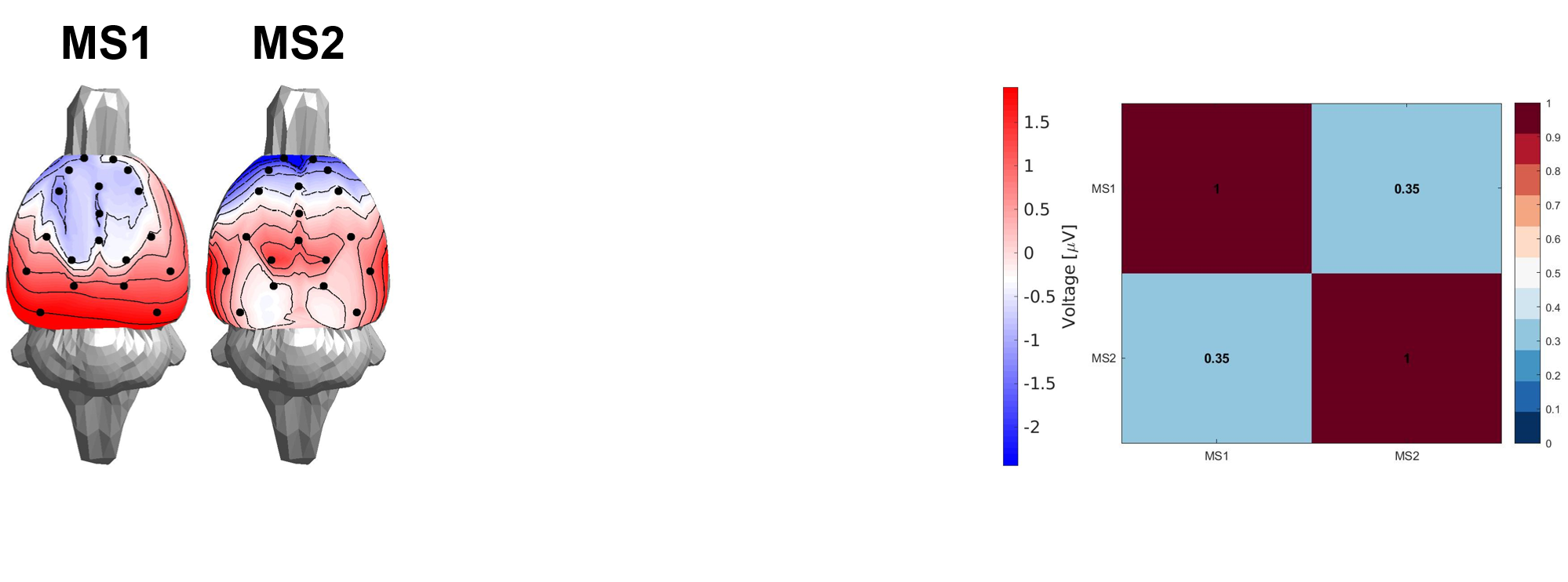


**Figure C1** *The topography of the microstate in the case of a two microstate total. The right panel defines the spatial correlation between the microstates.*


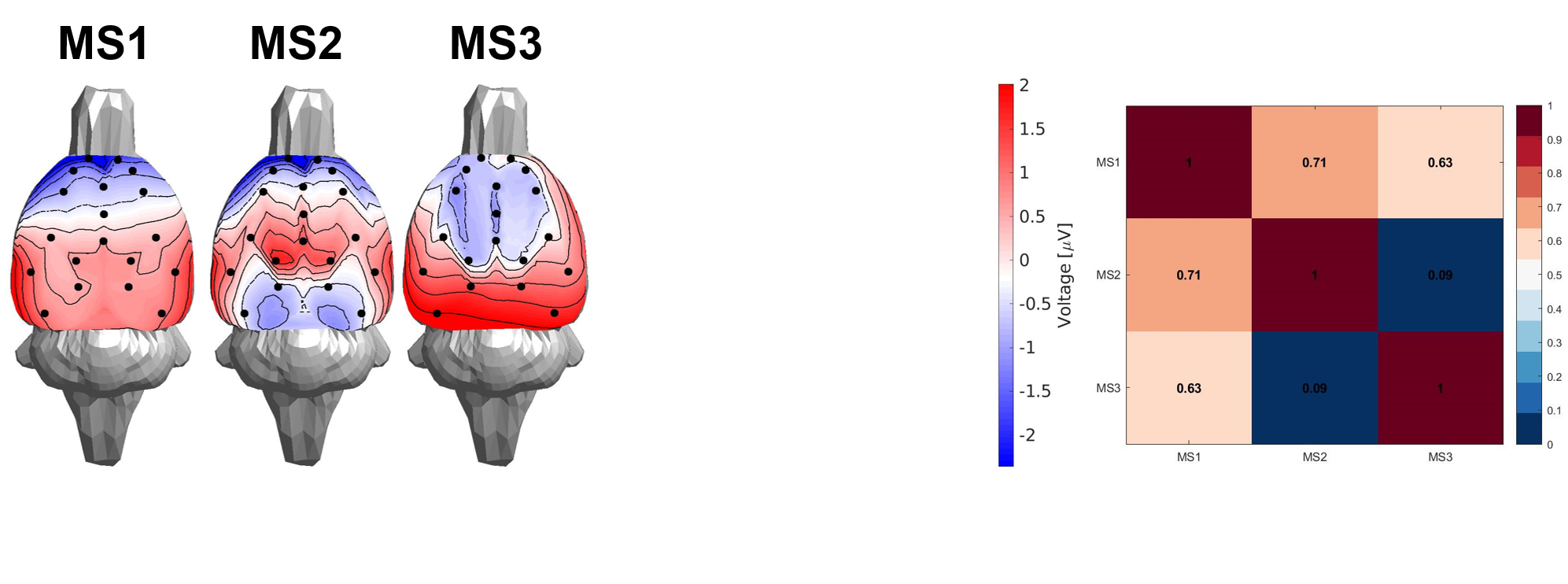


**Figure C2** *The topography of the microstate in the case of a three microstate total. The right panel defines the spatial correlation between the microstates.*


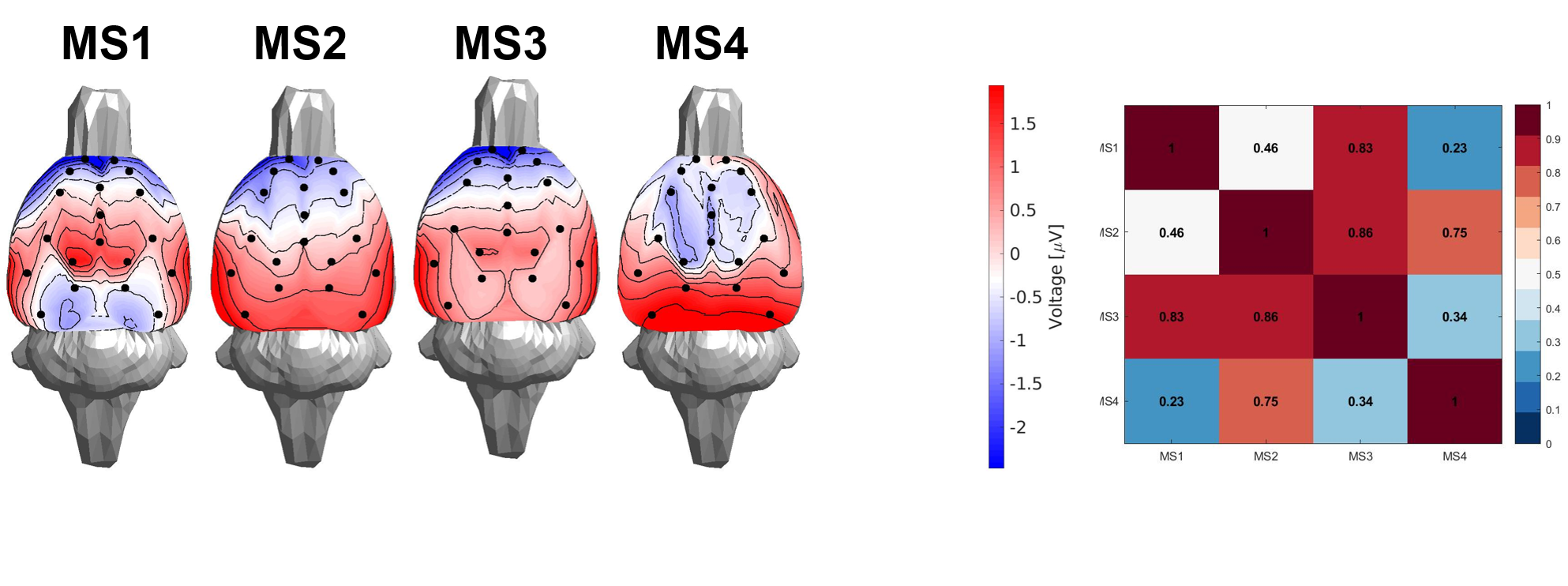


**Figure C3** *The topography of the microstate in the case of a four microstate total. The right panel defines the spatial correlation between the microstates.*


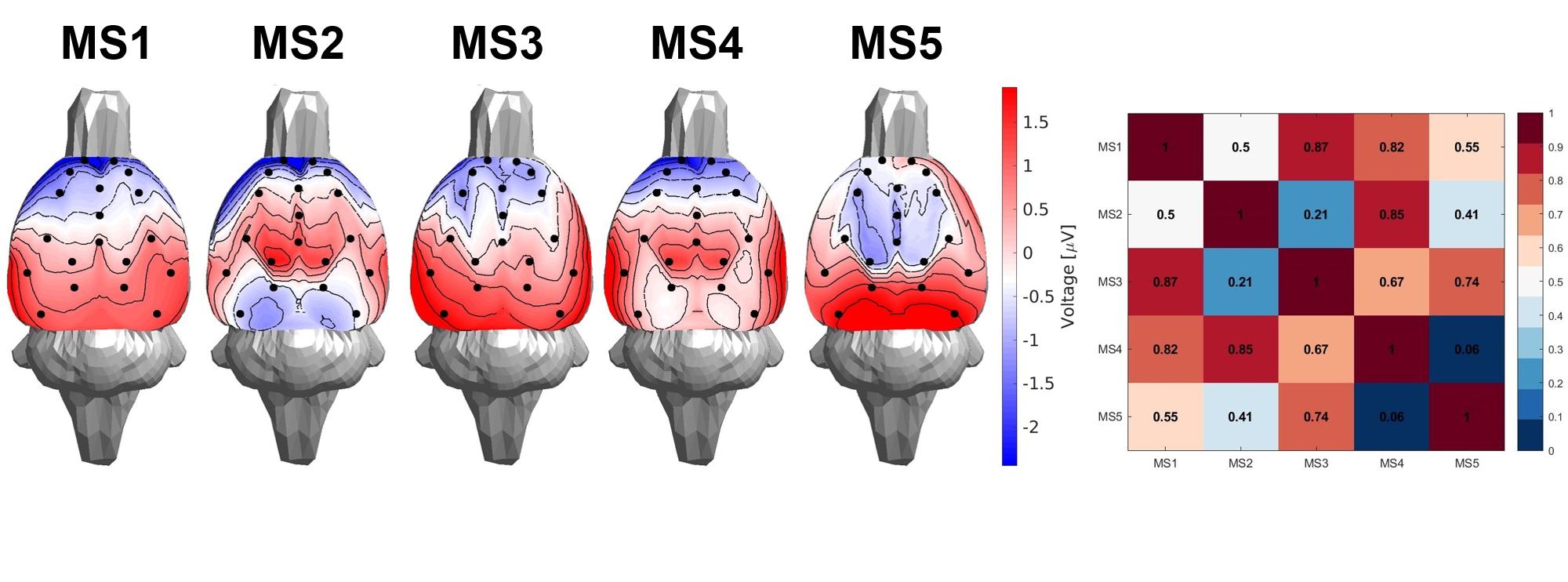


**Figure C4** *The topography of the microstate in the case of a five microstate total. The right panel defines the spatial correlation between the microstates.*


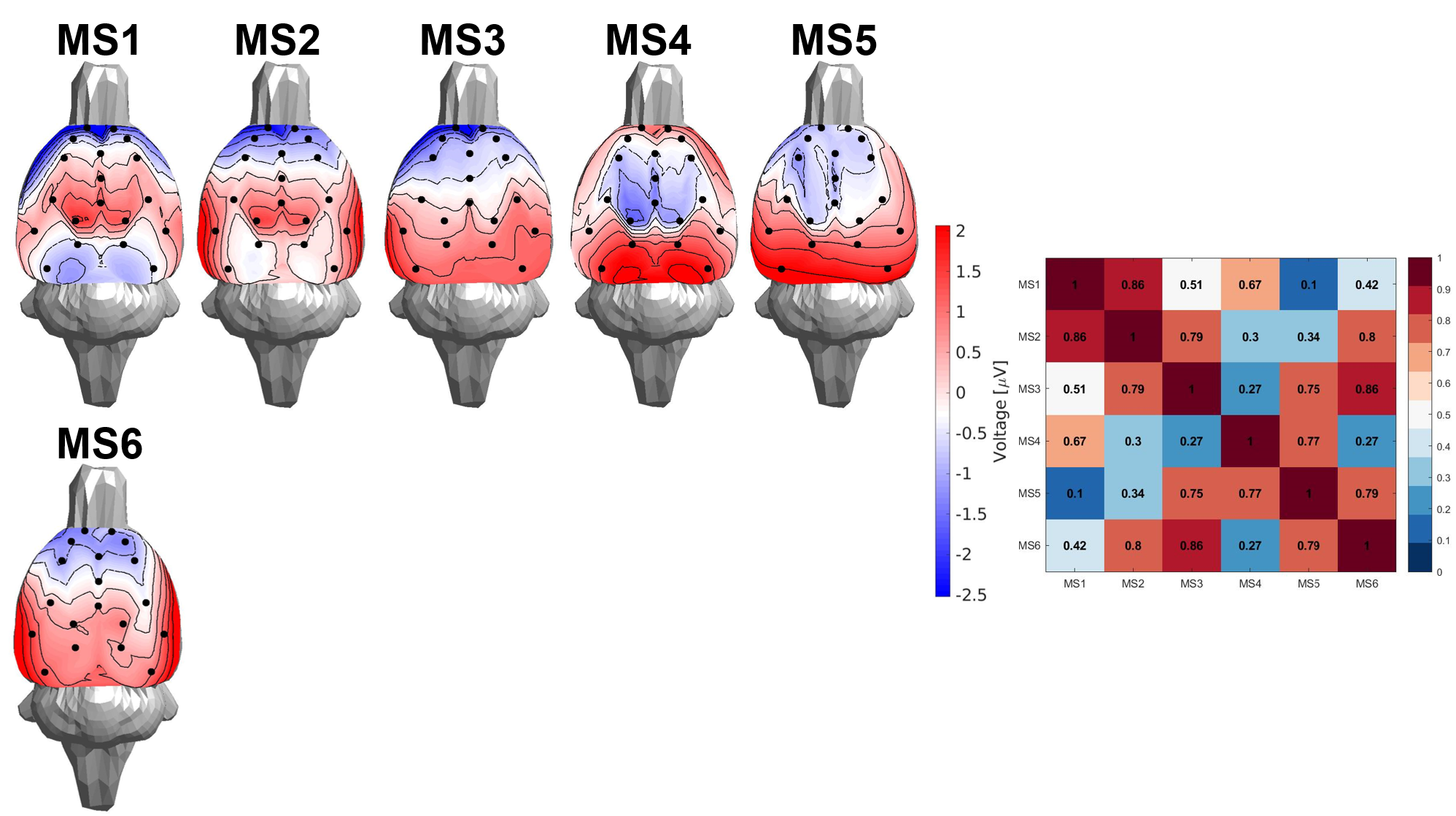


**Figure C5** *The topography of the microstate in the case of a six microstate total. The right panel defines the spatial correlation between the microstates.*


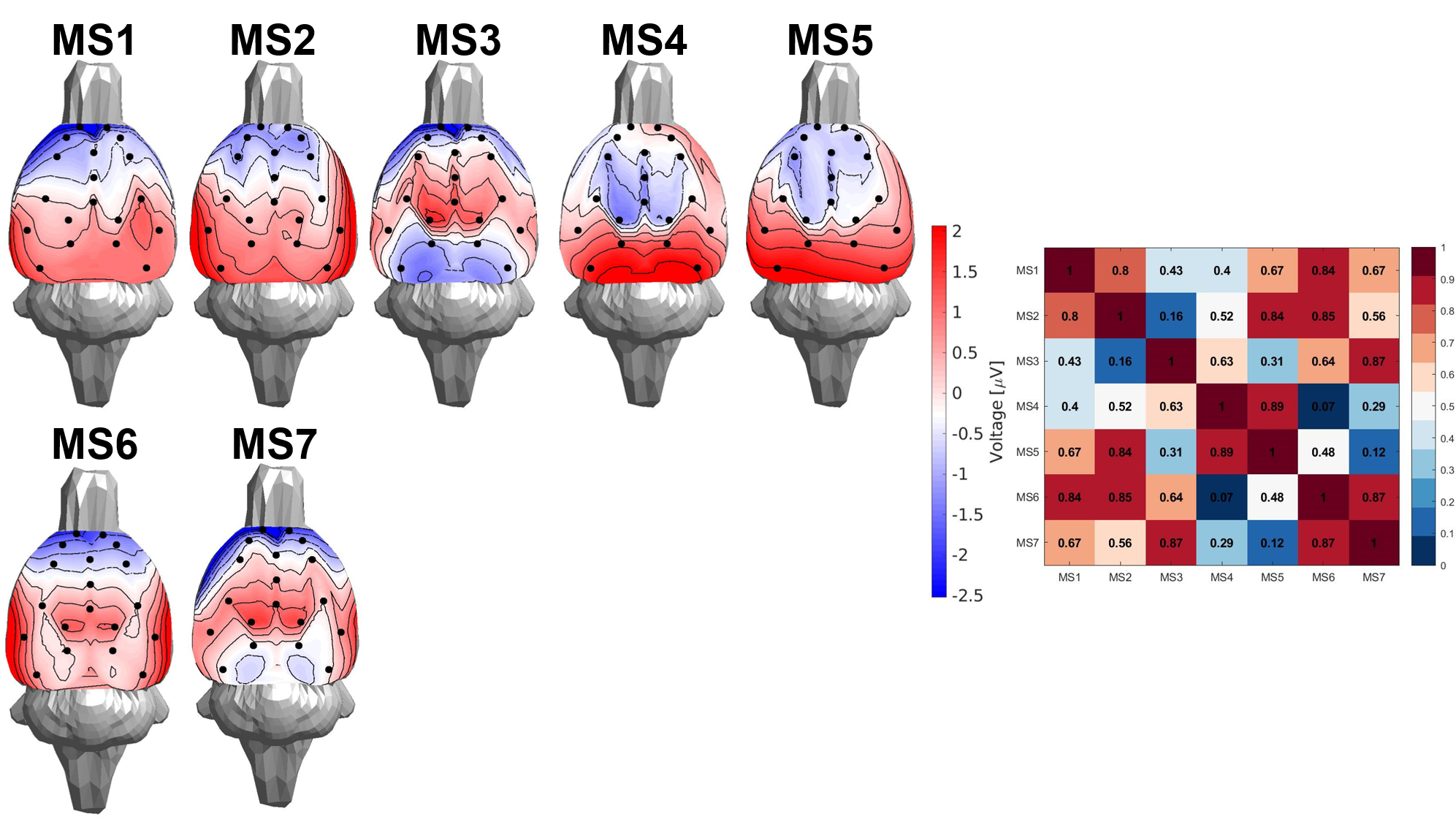


**Figure C6** *The topography of the microstate in the case of a seven microstate total. The right panel defines the spatial correlation between the microstates.*


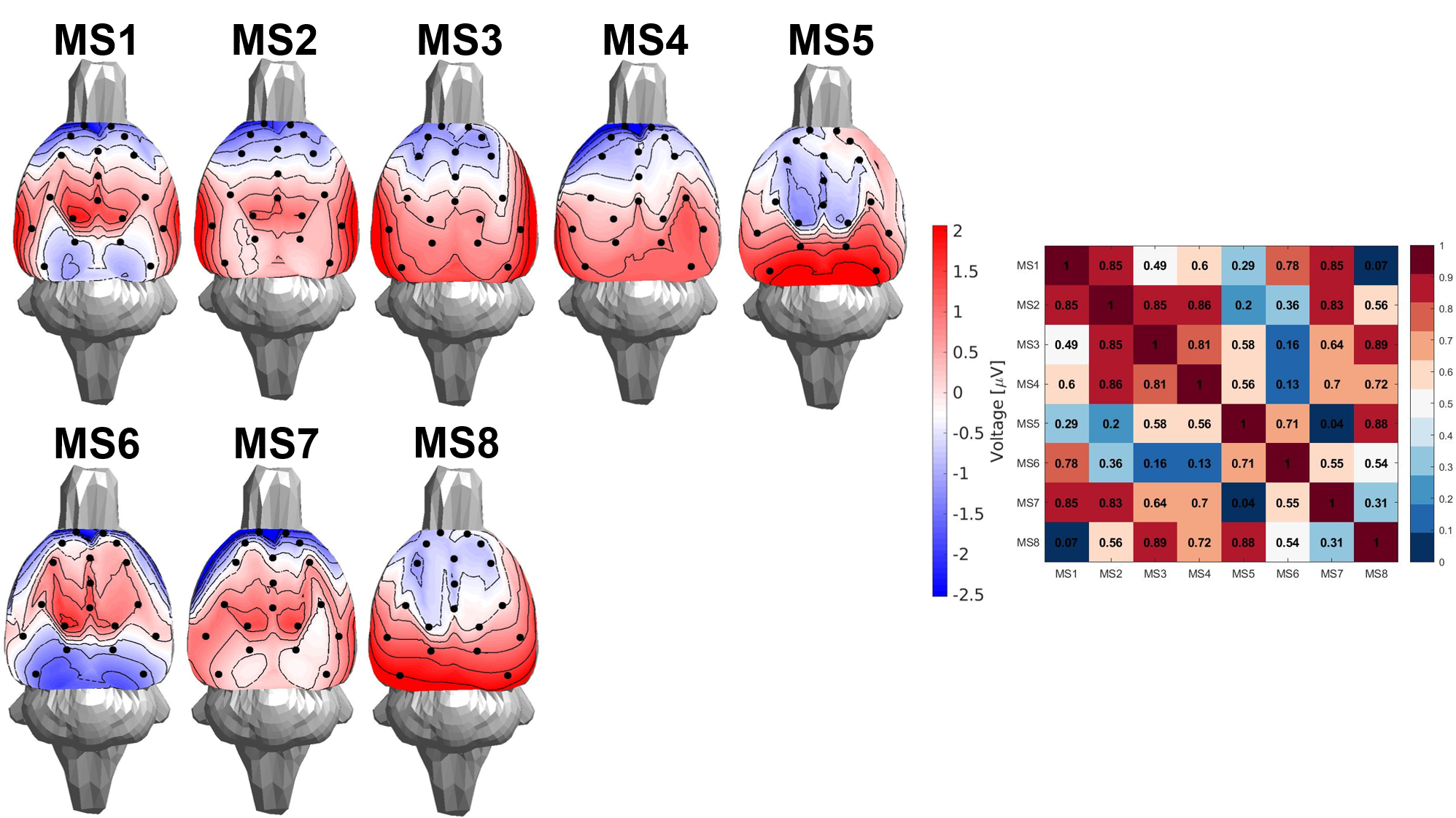


**Figure C7** *The topography of the microstate in the case of an eight microstate total. The right panel defines the spatial correlation between the microstates.*


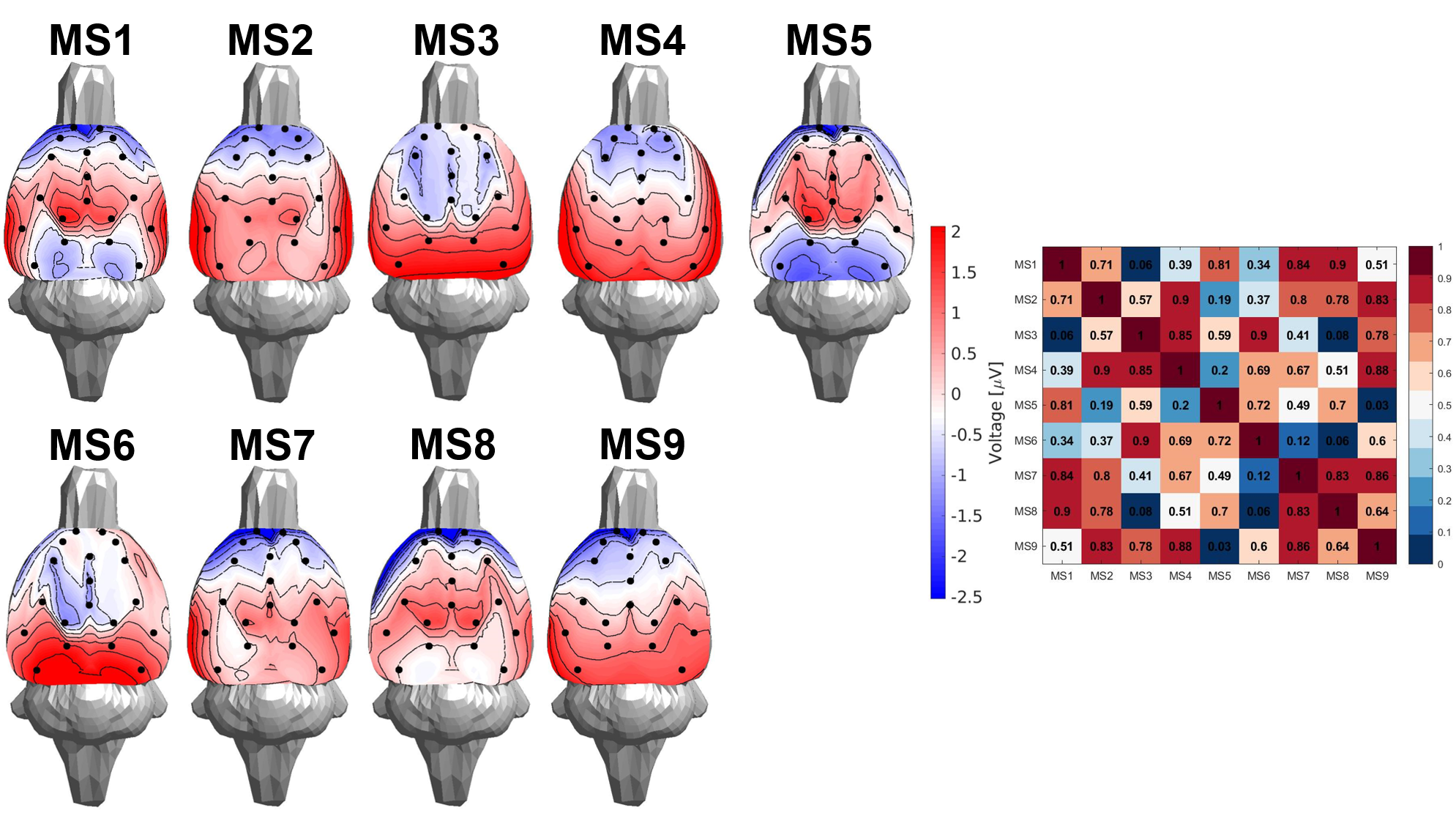


**Figure C8** *The topography of the microstate in the case of a nine microstate total. The right panel defines the spatial correlation between the microstates.*


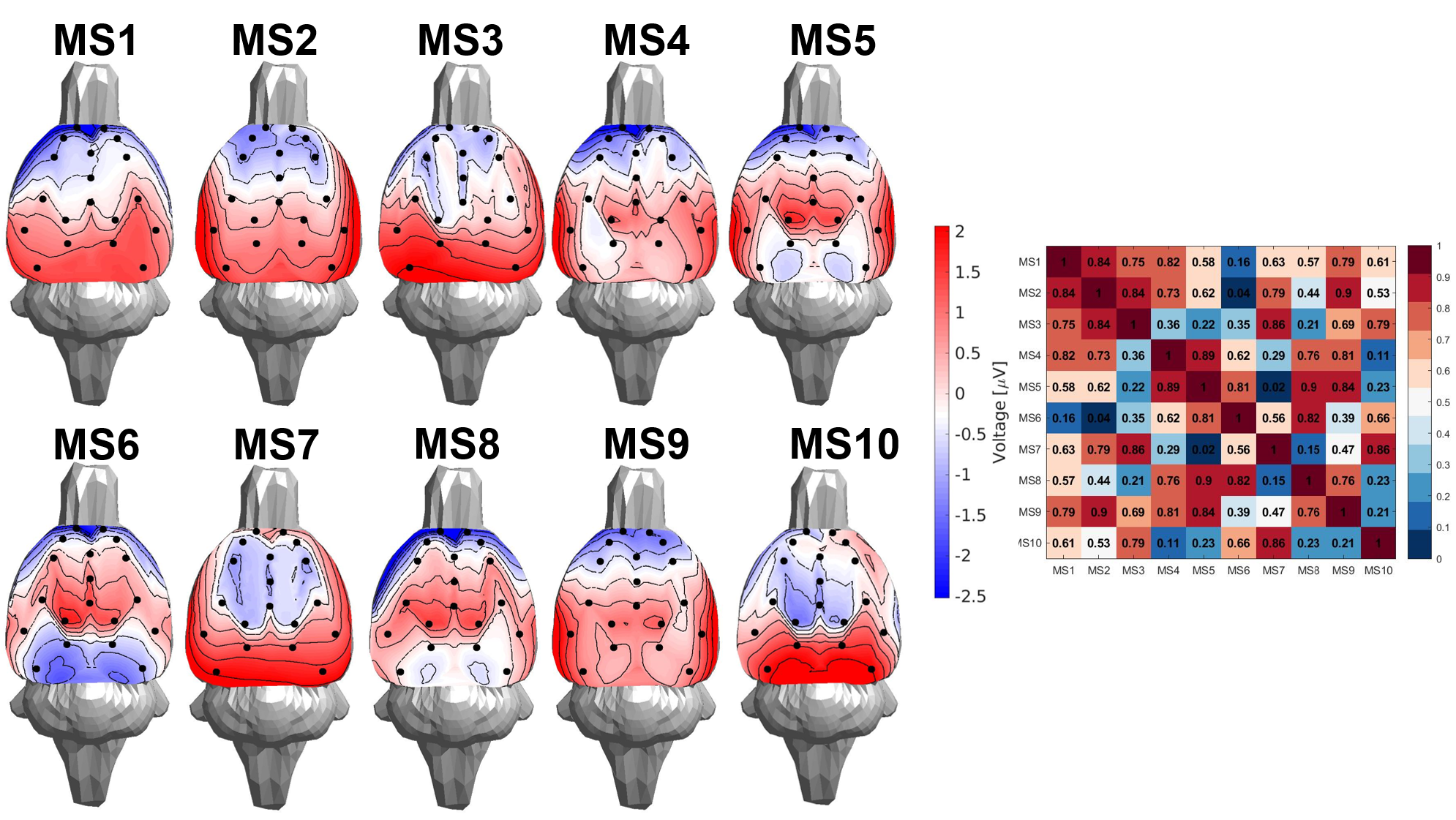


**Figure C9** *The topography of the microstate in the case of a ten microstate total. The right panel defines the spatial correlation between the microstates.*

# Appendix D Microstate Transition Probabilities

The observed and expected transition probabilities were computed and are presented in Figure D1, with their corresponding numerical values reported in Table D1. The differences between the observed and expected transitions were likewise calculated and are illustrated in Figure D2, with detailed values provided in Table D2.

To evaluate these differences, Mann–Whitney U tests were conducted, and significance levels were adjusted using a Bonferroni correction to account for the 20 possible transitions.


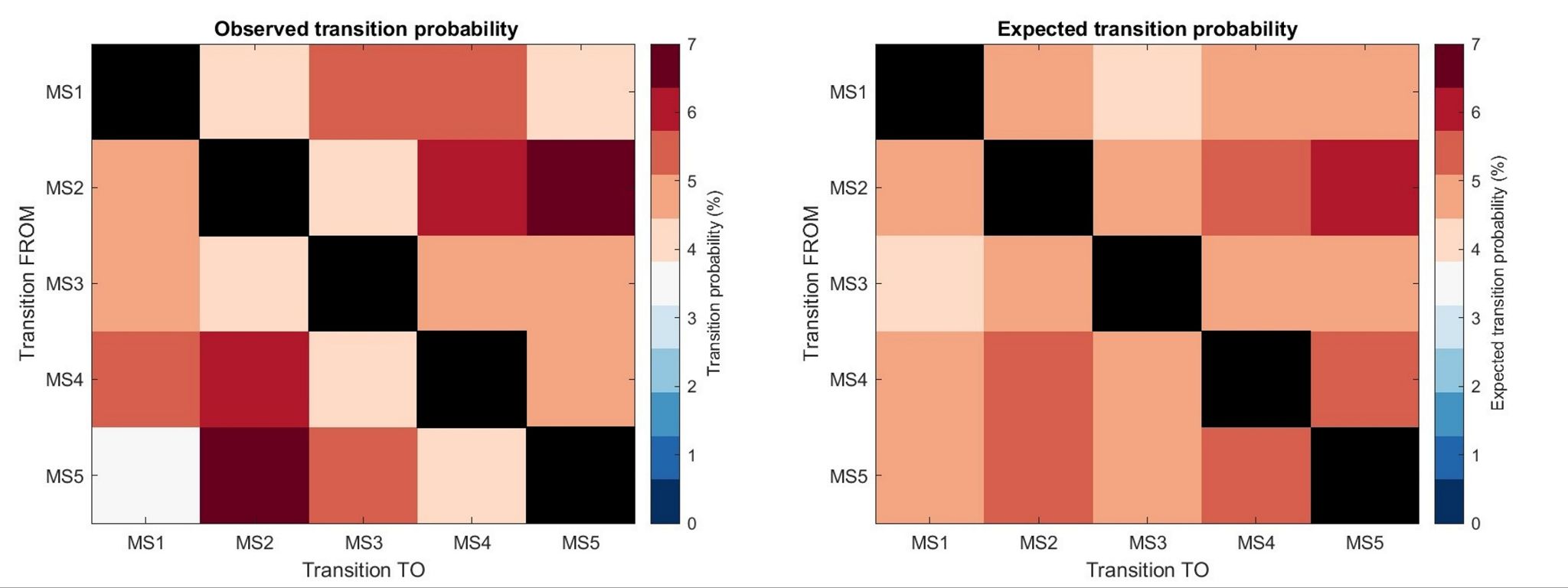


**Figure D1** *The observed (left) and expected transition probabilities (right).*

**Table D1**  *The observed/expected transition probabilities.*

| ***FROM \ TO*** | ***MS 1*** | ***MS 2*** | ***MS 3*** | ***MS 4*** | ***MS 5*** |
| --- | --- | --- | --- | --- | --- |
| ***MS 1*** | *NaN / NaN* | *4.00 / 4.93* | *5.26 / 4.42* | *5.21 / 4.64* | *4.03 / 4.53* |
| ***MS 2*** | *4.55 / 5.07* | *NaN / NaN* | *4.01 / 5.07* | *5.95 / 5.62* | *6.99 / 5.74* |
| ***MS 3*** | *5.02 / 4.42* | *4.24 / 4.91* | *NaN / NaN* | *4.74 / 4.80* | *5.09 / 4.95* |
| ***MS 4*** | *5.21 / 4.64* | *5.95 / 5.45* | *4.38 / 4.82* | *NaN / NaN* | *4.62 / 5.25* |
| ***MS 5*** | *3.74 / 4.64* | *7.31 / 5.68* | *5.44 / 5.07* | *4.25 / 5.35* | *NaN / NaN* |


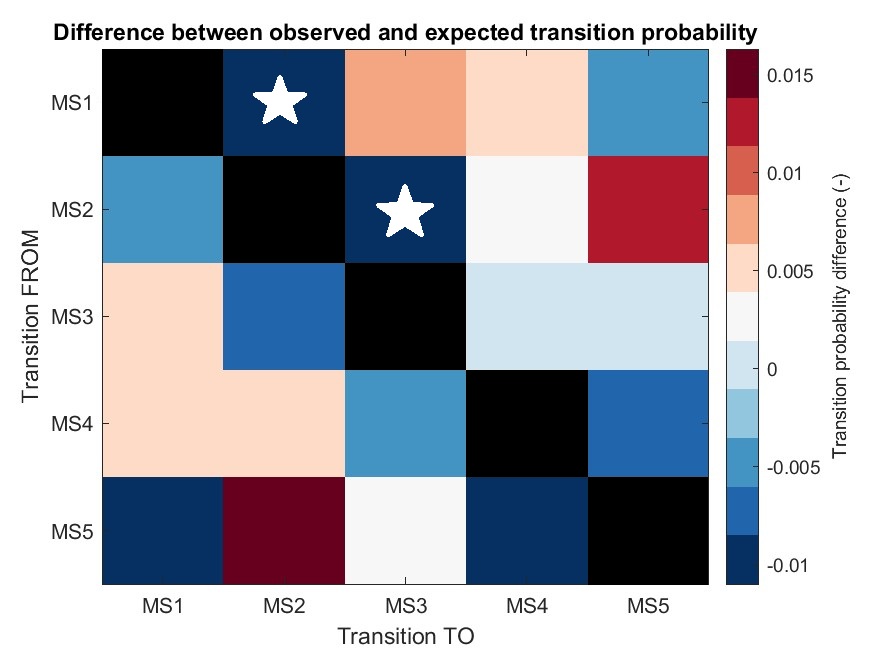


**Figure D2** *Significant differences between observed and expected probabilities (Mann–Whitney U test with Bonferroni correction). Asterisks denote significant comparisons.*

**Table D2** *The difference between the observed and expected probabilities. The asterisks represent the significant difference between the observed and expected probabilities.*

| **FROM \ TO** | **MS 1** | **MS 2** | **MS 3** | **MS 4** | **MS 5** |
| --- | --- | --- | --- | --- | --- |
| **MS 1** | NaN | -0.93* | 0.84 | 0.57 | -0.50 |
| **MS 2** | -0.52 | NaN | -1.06* | 0.33 | 1.25 |
| **MS 3** | 0.60 | -0.67 | NaN | -0.06 | 0.14 |
| **MS 4** | 0.57 | 0.50 | -0.44 | NaN | -0.63 |
| **MS 5** | -0.90 | 1.63 | 0.37 | -1.10 | NaN |

# References

[1] Custo, A., Van De Ville, D., Wells, W., Tomescu, M., Michel, C.: EEG resting-state networks: microstates’ source localization. Brain Connectivity 7 (2017). <https://doi.org/10.1089/brain.2016.0476>

[2] Davies, D.L., Bouldin, D.W.: A cluster separation measure. IEEE Transactions on Pattern Analysis and Machine Intelligence PAMI-1(2), 224–227 (1979). <https://doi.org/10.1109/TPAMI.1979.4766909>

[3] Krzanowski, W.J., Lai, Y.T.: A criterion for determining the number of groups in a data set using sum-of-squares clustering. Biometrics 44(1), 23–34 (1988). https://doi.org/10.2307/2531893

[4] Dunn, J.C.: Well-separated clusters and optimal fuzzy partitions. Cybernetics and Systems 4, 95–104 (1973). <https://doi.org/10.1080/01969727408546059>

[5] Charrad, M., Ghazzali, N., Boiteau, V., Niknafs, A.: Nbclust: An r package for determining the relevant number of clusters in a data set. Journal of Statistical Software 61(6), 1–36 (2014). <https://doi.org/10.18637/jss.v061.i06>

[6] Frey, T., van Groenewoud, H.: A cluster analysis of the d2 matrix of white spruce stands in saskatchewan based on the maximum-minimum principle. Journal of Ecology 60(3), 873–886 (1972). https://doi.org/10.2307/2258571

[7] Poulsen, A., Pedroni, A., Langer, N., Hansen, L.: Microstate EEGLAB toolbox: An introductory guide (2018). <https://doi.org/10.1101/289850>

[8] Pascual-Marqui, R.D., Michel, C.M., Lehmann, D.: Segmentation of brain electrical activity into microstates: model estimation and validation. IEEE Transactions on Biomedical Engineering 42(7), 658–665 (1995). <https://doi.org/10.1109/10.391164>

[9] Murray, M., Brunet, D., Michel, C.: Topographic erp analyses: A step-by-step tutorial review. Brain topography 20, 249–64 (2008). [https://doi. org/10.1007/s10548-008-0054-5](https://doi)
